# Beyond readthrough: ataluren restores mitochondrial function and reduces oxidative stress in *FANCA*-mutated cells via mTOR–DRP1 modulation

**DOI:** 10.1038/s41420-026-02983-6

**Published:** 2026-02-28

**Authors:** Matilde Balbi, Elisa Guidi, Anca Manuela Hristodor, Fabio Corsolini, Vanessa Cossu, Roberta Bottega, Martina Serra, Sara Pestarino, Martina Bartolucci, Marco Cipolli, Stefano Regis, Valentino Bezzerri, Enrico Cappelli, Silvia Ravera

**Affiliations:** 1 Department of Experimental Medicine, University of Genoa, Genoa, Italy; 2 Cystic Fibrosis Center, Azienda Ospedaliera Universitaria Integrata, Verona, Italy; 3 Unit of Hematology, IRCCS Istituto Giannina Gaslini, Genoa, Italy; 4 IRCCS Ospedale Policlinico San Martino, Genoa, Italy; 5 Institute for Maternal and Child Health, IRCCS Burlo Garofolo, Trieste, Italy; 6 Department of Neurosciences, Rehabilitation, Ophthalmology, Genetics, and Maternal and Child Health, University of Genoa, Genoa, Italy; 7 Hematopoietic Stem Cell Transplantation Unit, Department of Pediatric Hematology-Oncology, IRCCS Istituto Giannina Gaslini, Genoa, Italy; 8 Clinical Proteomics and Metabolomics Core Facility, IRCCS Istituto Giannina Gaslini, Genoa, Italy; 9 UOSD Laboratorio di Terapie Cellulari, IRCCS Istituto Giannina Gaslini, Genova, Italy; 10 Department of Life Sciences, Health and Health Professions, Link Campus University, Rome, Italy

**Keywords:** Cell biology, Biochemistry

## Abstract

Fanconi anemia (FA) is a rare inherited bone marrow failure syndrome characterized by genomic instability, mitochondrial dysfunction, and oxidative stress. While the therapeutic potential of ataluren, a translational readthrough-inducing drug, has been investigated in FA cells carrying nonsense mutations, its broader metabolic impact remains unclear. Here, we demonstrate that ataluren (tested at 2.5, 5, and 10 μM) modulates cellular energy metabolism and redox homeostasis in FA lymphoblasts harboring either nonsense or missense mutations in the *FANCA* gene. At low doses (2.5 μM for 72 h), ataluren improved the ATP/AMP ratio, enhanced oxidative phosphorylation efficiency, and reduced lipid peroxidation and oxidative DNA damage. These effects were independent of mutation type and were not associated with compensatory glycolysis, as lactate dehydrogenase activity remained unchanged. Strikingly, ataluren restored the P/O ratio under pyruvate/malate-driven respiration to near-normal values, indicating improved coupling between oxygen consumption and ATP synthesis. Mechanistically, ataluren reduced DRP1 protein levels and attenuated mTOR-S6 signaling, suggesting that mitochondrial dynamics and bioenergetic efficiency are modulated via the mTOR–DRP1 axis. Additionally, ataluren lowered IMPDH activity, contributing to reduced cell proliferation and DNA damage without impairing cellular energy status. Notably, these beneficial effects persisted under immune stimulation, where ataluren mitigated the metabolic and oxidative burden imposed by lymphocyte activation. Our findings unveil a pleiotropic role for ataluren that extends beyond its canonical readthrough activity, highlighting its potential as a metabolic modulator for FA and possibly other DNA repair–deficient disorders.

## Introduction

Approximately 11% of human genetic diseases are caused by nonsense mutations that introduce premature stop codons within the open reading frame (ORF) [1], leading to early termination of translation and the production of truncated, non-functional proteins [1, 2]. The release of the nascent peptide and disassembly of the translation complex are orchestrated by a heterodimer composed of eukaryotic release factors eRF1 and eRF3 [3]. Specifically, eRF1 recognizes all three stop codons (UAA, UAG, UGA) [3], while eRF3 mediates GTP-dependent hydrolysis, enabling peptide release and ribosome recycling [3].

However, sometimes, ribosomes bypass premature stop codons, resulting in the incorporation of an amino acid via a near-cognate tRNA and continuation of translation through a process known as translation readthrough [4, 5]. This mechanism often results in aberrant proteins, although, under certain conditions, it can restore the expression of full-length functional proteins, giving protein isoforms [5]. Therapeutically, translation readthrough has been explored to address genetic disorders characterized by high frequencies of nonsense mutations [6]. In this view, several small molecules, such as aminoglycosides, seem able to overcome premature terminal codons by inserting near-cognate tRNAs, allowing ribosomes to synthesize full-length protein [2]. Due to its good safety profile, ataluren, a benzoic acid oxadiazole, was conditionally approved for clinical use in Duchenne muscular dystrophy (DMD) [7]. Ataluren can induce readthrough of all nonsense codons, with higher affinity for UGA [8], acting by competitively binding to two sites on rRNA and one on eRF1, thus interfering with the nonsense codons recognition [9]. In addition, ataluren does not show side effects on mRNA translation, does not alter mRNA level and stability, and does not induce transcriptional alterations [10]. Recently, ataluren has been proposed as a candidate therapeutic agent for Inherited Bone Marrow Failure Syndromes (IBMFS) [11], a group of genetically heterogeneous disorders characterized by impaired hematopoiesis and often linked to defects in DNA repair or ribosome biogenesis [12]. In Shwachman-Diamond syndrome (SDS), where nonsense mutations in the SBDS gene are found in approximately 55% of patients [13], preclinical models have shown that ataluren restores SBDS protein expression, improving both myelopoiesis and neutrophil chemotaxis [13]. More recently, attention has turned to Fanconi anaemia (FA), an IBMFS caused by biallelic mutations in genes involved in the FA/BRCA DNA repair pathway [14]. FA cells display hallmark features including chromosomal instability, hypersensitivity to DNA crosslinking agents, and defective homologous recombination repair [15, 16]. Beyond DNA repair dysfunction, studies have highlighted a profound metabolic reprogramming in FA cells [17, 18]. Specifically, mitochondrial fragmentation, impaired oxidative phosphorylation (OxPhos), and altered lipid metabolism have been observed [19–23]. These cells exhibit elevated levels of reactive oxygen species (ROS), an altered redox state, and a failure to adequately respond to oxidative stress [24, 25]. Additionally, pro-inflammatory cytokine dysregulation contributes to bone marrow failure and immune dysfunction in FA, promoting cellular senescence [26, 27]. Since treatment with ataluren reduced the spontaneous and induced double-strand breaks (DSBs) in FA cells [28], we explored whether ataluren could also influence the energy and redox metabolism in FA cells carrying homozygous nonsense mutations in *FANCA*, the most frequently mutated FA gene. Intriguingly, treatment with ataluren significantly improved mitochondrial function, enhancing OxPhos efficiency, restoring ATP levels, and reducing oxidative stress markers, not only in FA cells with nonsense mutation of the *FANCA* gene but also in cells carrying missense mutations, suggesting that ataluren may exert pleiotropic effects on cellular metabolism beyond its canonical role in promoting translational readthrough.

## Results

### Ataluren modulates cellular energy status in FANCA-mutated lymphoblasts regardless of the presence of nonsense mutations

To investigate whether ataluren can influence this bioenergetic imbalance typical of FA cells [21], we assessed the effects of different concentrations of drug (2.5, 5, and 10 μM) over time (24, 48, and 72 h (h) post-treatment) on intracellular ATP and AMP levels in lymphoblasts derived from FA patients harboring either nonsense or missense mutations in the *FANCA* gene. Results were compared to those obtained in lymphoblasts from healthy donors.

**Fig. 1:**
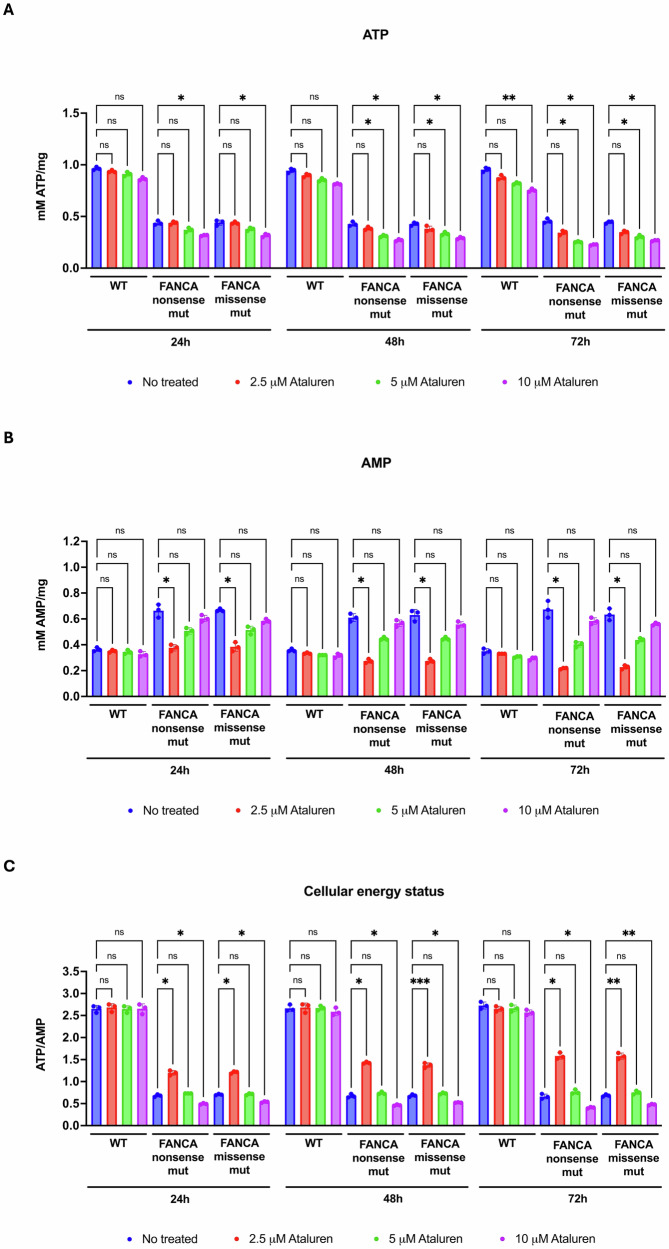
Intracellular ATP and AMP levels and cellular energy status in FANCA lymphoblasts treated with ataluren. Analyses were performed on lymphoblasts isolated from a healthy donor (WT) and from FA patients carrying nonsense or missense mutations (mut). Ataluren treatment was applied for 24, 48, and 72 h at three different concentrations: 2.5, 5, and 10 µM. **A** Intracellular ATP content. **B** Intracellular AMP content. **C** Cellular energy status, calculated as the ATP/AMP ratio. Data are presented as mean ± SD and are representative of three independent experiments (*n* = 3). *, **, and *** indicate statistically significant differences at *p* < 0.05, 0.01, and 0.001, respectively. ns indicates no statistically significant difference.

Our data show that in both nonsense and missense *FANCA*-mutated lymphoblasts, treatment with ataluren at 5 and 10 μM leads to a dose-dependent reduction in intracellular ATP levels (Fig. 1A). In contrast, treatment with the lowest tested concentration (2.5 μM) did not produce significant changes in ATP intracellular concentration compared to untreated cells, even after 72 h. Conversely, ataluren exhibited a hormetic effect on intracellular AMP levels (Fig. 1B) at 2.5 μM, a decrease in AMP levels was observed over time in both nonsense and missense mutant lymphoblasts, whereas higher concentrations (5 and 10 μM) induced a downward trend that did not reach statistical significance relative to untreated controls.

These distinct effects on ATP and AMP levels translated into differential impacts on cellular energy status, as reflected by the ATP/AMP ratio (Fig. 1C). Specifically: (i) ataluren at 2.5 μM induced a time-dependent increase in the ATP/AMP ratio compared to untreated cells (for nonsense mutations: 76.11 ± 8.48% increase at 24 h vs. 140.05 ± 13.69% at 72 h, p 24 h vs 72 h = 0.0023; for missense mutations: 72.15 ± 7.42% at 24 h vs. 130.78 ± 8.03% at 72 h, p 24 h vs 72 h = 0.0007); (ii) 5 μM treatment had no significant effect on the ATP/AMP ratio at any time point; and (iii) 10 μM treatment resulted in a time-dependent reduction in the ATP/AMP ratio (for nonsense mutations: 27.42 ± 1.10% reduction at 24 h vs. 38.22 ± 3.02% at 72 h, p 24 h vs 72 h = 0.0043; for missense mutations: 23.67 ± 2.95% reduction at 24 h vs. 30.23 ± 0.79% at 72 h, p 24 h vs 72 h = 0.0203).

Notably, the effects of ataluren on cellular energy status appeared to be independent of the mutation type in the FANCA gene, since ATP and AMP concentrations, as well as their ratio, were modulated in a similar manner in both nonsense- and missense-mutated cells, despite in nonsense-mutated cells, ataluren slightly increased the expression of the FANCA protein as reported in [28]. This suggests that ataluren modulates the cellular energy balance in *FANCA*-mutant lymphoblasts regardless of the specific mutational profile. In addition, lymphoblasts derived from healthy donors did not show any response to ataluren treatment, except for a slight decrease in ATP levels after 72 h at the highest dose (10 µM).

### Ataluren enhances oxidative phosphorylation efficiency by attenuating its activity but does not affect anerobic glycolysis in FANCA-mutated lymphoblasts regardless of the presence of nonsense mutations

**Fig. 2:**
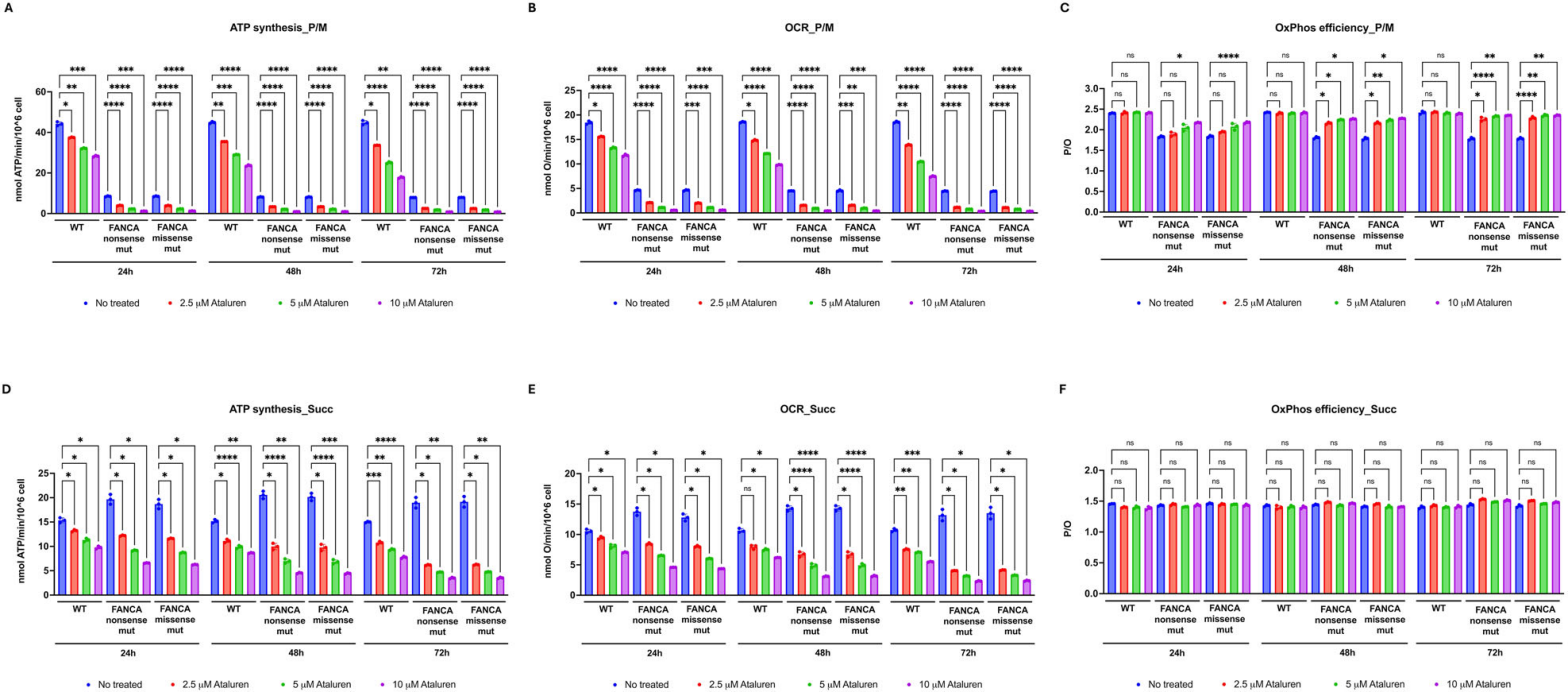
OxPhos function and efficiency in FANCA lymphoblasts treated with ataluren. Analyses were performed on lymphoblasts isolated from a healthy donor (WT) and from FA patients carrying nonsense or missense mutations (mut). Ataluren treatment was applied for 24, 48, and 72 h at three different concentrations: 2.5, 5, and 10 µM. **A** ATP synthesis through FoF₁-ATP synthase in the presence of pyruvate plus malate (P/M). **B** Oxygen consumption rate (OCR) in the presence of pyruvate/malate (P/M). **C** P/O value, a marker of OxPhos efficiency, in the presence of P/M. **D** ATP synthesis through FoF₁-ATP synthase in the presence of succinate (Succ). **E** OCR in the presence of succinate (Succ). **F** P/O value in the presence of Succ. Data are presented as mean ± SD and are representative of three independent experiments (*n* = 3). *, **, ***, and **** indicate statistically significant differences at *p* < 0.05, 0.01, 0.001, and 0.0001, respectively. ns indicates no statistically significant difference.

Given that the energy deficit observed in *FANCA*-mutated cells depends on impaired OxPhos function [20], aerobic ATP synthesis, oxygen consumption rate (OCR), and the corresponding P/O ratio were evaluated in FA lymphoblasts carrying either nonsense or missense mutations in the presence of ataluren. As expected, cells carrying either the nonsense or missense *FANCA* mutation exhibit marked dysfunction in both ATP synthesis (Fig. 2A) and OCR (Fig. 2B) when stimulated with pyruvate/malate (P/M), resulting in an altered P/O ratio (Fig. 2C). This confirms a defect in electron transfer between Complex I and Complex III [20]. In contrast, when succinate (Succ) was used as a substrate, *FANCA*-mutated cells display higher ATP synthesis and respiration compared to cells derived from healthy donors, with whom they share full OxPhos efficiency through the Complex II–driven pathway (Fig. 2D, E, F, respectively). Ataluren treatment caused a reduction in both aerobic ATP production (Fig. 2A, D) and OCR (Fig. 2B, E), both in healthy donor-derived lymphoblasts as well as in *FANCA*-mutant cells. This effect was evident as early as 24 h following treatment with the lowest dose tested (2.5 μM), and it became more pronounced after 72 h of exposure to the highest dose (10 μM) (Supplementary Table I). The reduction in OxPhos activity was observed under both P/M- and Succ-driven conditions, indicating that ataluren modulates both the pathways involved in OxPhos.

The most striking finding concerns the behavior of the P/O ratio, a direct indicator of OxPhos efficiency (Fig. 2C, F). In healthy lymphoblasts, the expected reference values for proper coupling between ATP synthesis and oxygen consumption (2.5 with P/M and 1.5 with Succ) remained stable under all conditions. Similarly, in *FANCA*-mutated cells, mitochondrial efficiency in the presence of Succ remained unchanged (Fig. 2F). In contrast, the reduced P/O value in *FANCA*-mutated cells under P/M stimulation was improved by ataluren treatment, reaching near-normal levels after 72 h—even at the lowest tested concentration (2.5 μM)—in both nonsense and missense mutation carriers (Fig. 2C and Supplementary Table I).

**Fig. 3:**
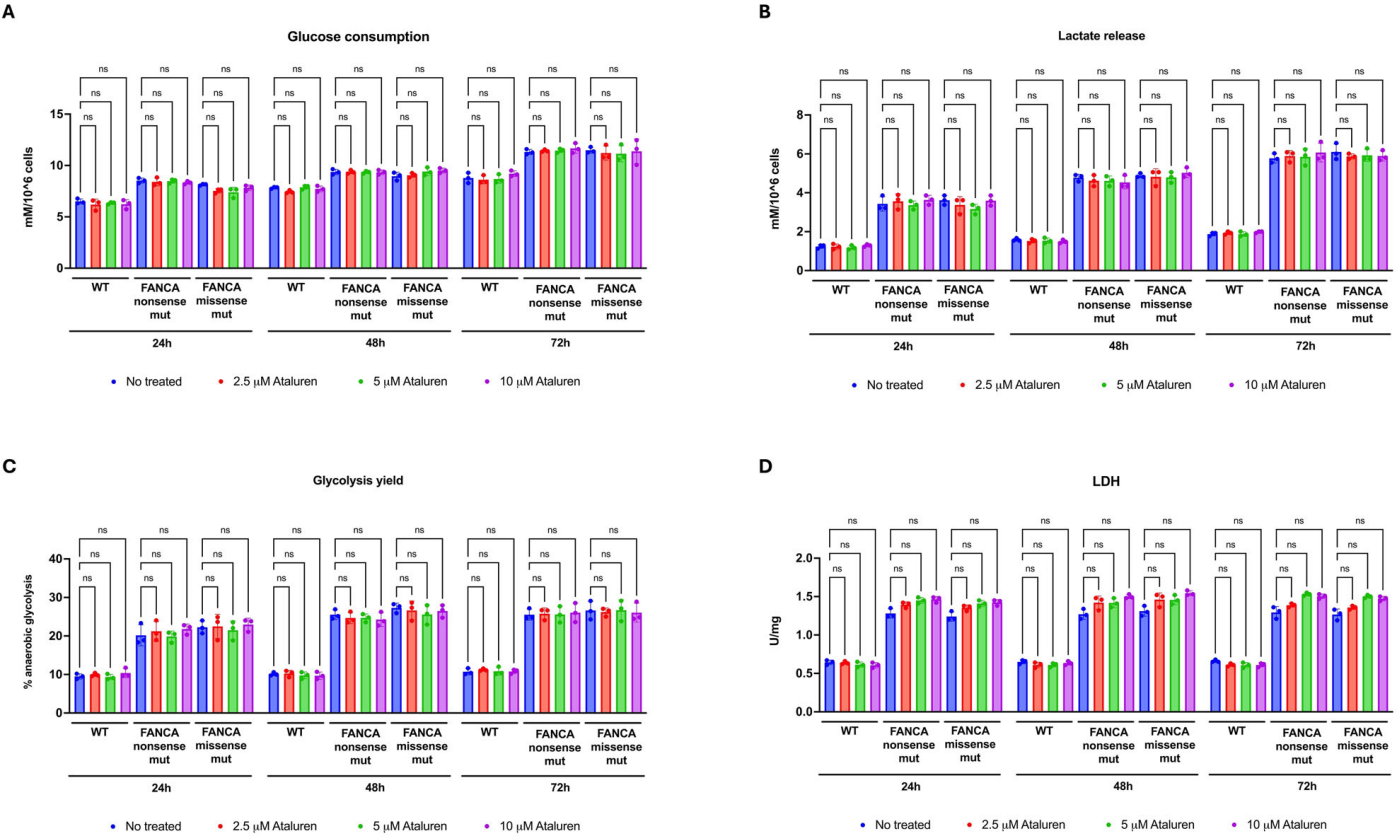
LDH activity in FANCA lymphoblasts treated with ataluren. Analyses were performed on lymphoblasts isolated from a healthy donor (WT) and from FA patients carrying nonsense or missense mutations (mut). Ataluren treatment was applied for 24, 48, and 72 h at three different concentrations: 2.5, 5, and 10 µM. **A** Glucose consumption. **B** Lactate release. **C** Glycolysis yield. **D** lactate dehydrogenase (LDH) activity. Data are presented as mean ± SD and are representative of three independent experiments (*n* = 3). ns indicates no statistically significant difference.

Interestingly, ataluren treatment did not affect glucose consumption, lactate release, or anaerobic glycolysis yield (Fig. 3A, B, C, respectively), nor lactate dehydrogenase (LDH) activity, which in FANCA-mutated lymphoblasts remained statistically comparable between ataluren-treated and untreated samples (Fig. 3D), despite a slight upward trend. This indicates that the reduction in OxPhos activity does not lead to a compensatory increase in anaerobic glycolysis and suggests that the rescue of the cellular energy status may depend on other mechanisms.

### Ataluren reduces lipid peroxidation and oxidative DNA damage in FANCA-mutated lymphoblasts regardless of the presence of nonsense mutations

To confirm that the attenuation of OxPhos activity, along with the concurrent improvement in its efficiency, contributes to mitigating oxidative damage—one of the hallmarks of *FANCA*-mutated cells [29]—levels of malondialdehyde (MDA) and 8-hydroxy-2’-deoxyguanosine (8OHdG) were measured as markers of lipid peroxidation and oxidative DNA damage, respectively, in both nonsense and missense *FANCA*-mutant lymphoblasts.

**Fig. 4:**
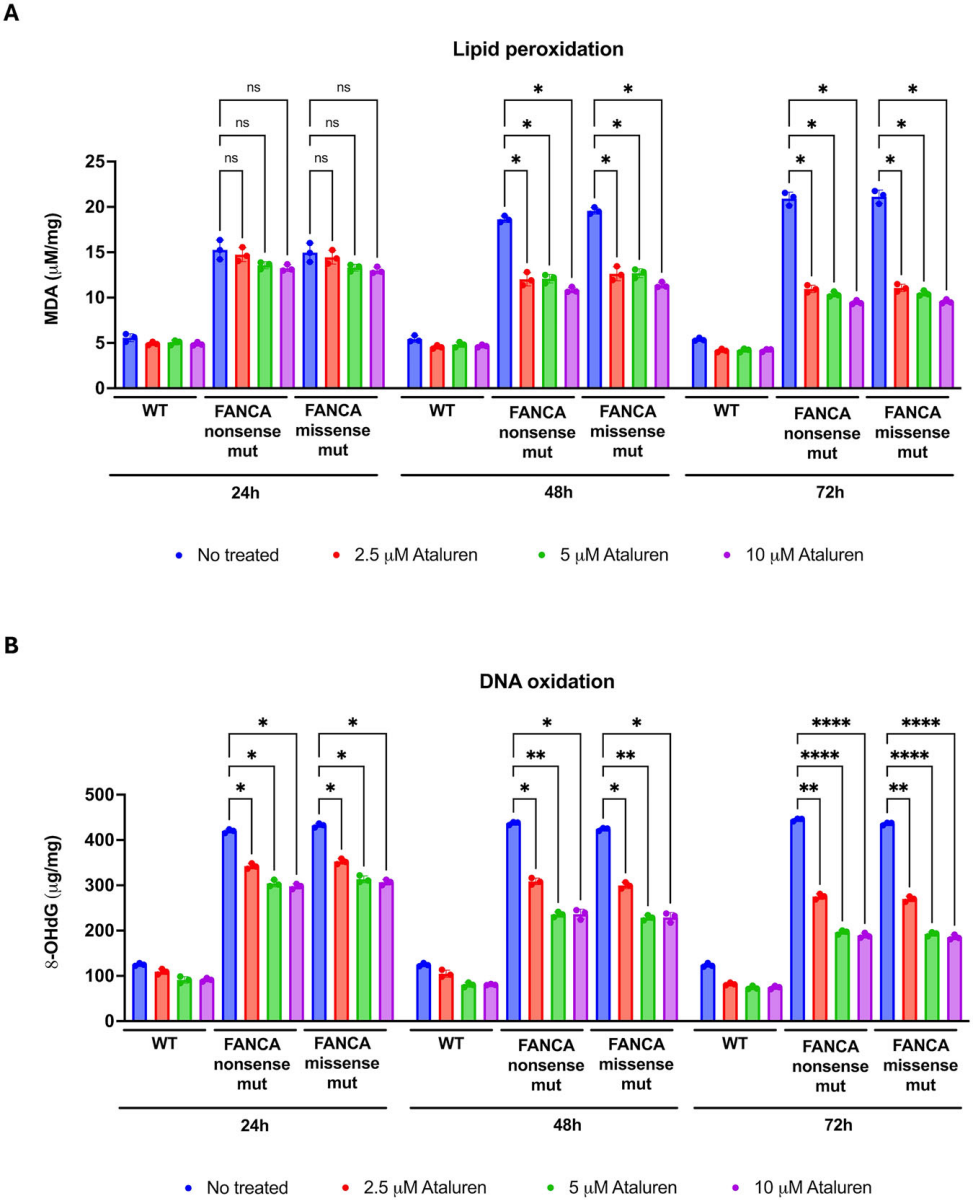
Oxidative damage in FANCA lymphoblasts treated with ataluren. Analyses were performed on lymphoblasts isolated from a healthy donor (WT) and from FA patients carrying nonsense or missense mutations (mut). Ataluren treatment was applied for 24, 48, and 72 h at three different concentrations: 2.5, 5, and 10 µM. **A** Malondialdehyde (MDA) intracellular concentration is a lipid peroxidation marker. **B** 8-hydroxy-2’-deoxyguanosine (8-OHdG) content as a DNA oxidation marker. Data are presented as mean ± SD and are representative of three independent experiments (*n* = 3). ns indicates no statistically significant difference.

As shown in Fig. 4A, ataluren treatment at all three tested concentrations did not significantly alter MDA levels within the first 24 h, although a decreasing trend was observed. However, at both 48 and 72 h, a dose-dependent reduction in MDA levels was detected compared to untreated cells, even though the values remained higher than those observed in lymphoblasts from healthy donors.

Conversely, 8OHdG levels decreased as early as 24 h post-treatment in *FANCA*-mutated cells, with the most pronounced reduction observed at 72 h (Fig. 4B). Notably, this effect was evident in both nonsense and missense mutant cell lines.

In light of the results described thus far, it is evident that the effect of ataluren on the metabolic alterations and oxidative damage characteristic of FA cells is not dependent on the recovery of FANCA protein expression, as the ataluren effects are overlapped in lymphoblasts carrying either nonsense or missense *FANCA* mutations. Therefore, the subsequent experiments were conducted exclusively in lymphoblasts harboring a missense mutation in the *FANCA* gene to investigate the mechanisms underlying ataluren-induced metabolic modulation.

Moreover, considering the combined effects of the three tested ataluren concentrations and the different time points of observation, we determined that the optimal condition for achieving a simultaneous improvement in the ATP/AMP ratio, OxPhos efficiency, and reduction in oxidative damage was treatment with 2.5 μM ataluren for 72 h.

### Ataluren improves cellular energy status and reduces oxidative damage in FA cells under basal conditions and following R848-induced immune activation

**Fig. 5:**
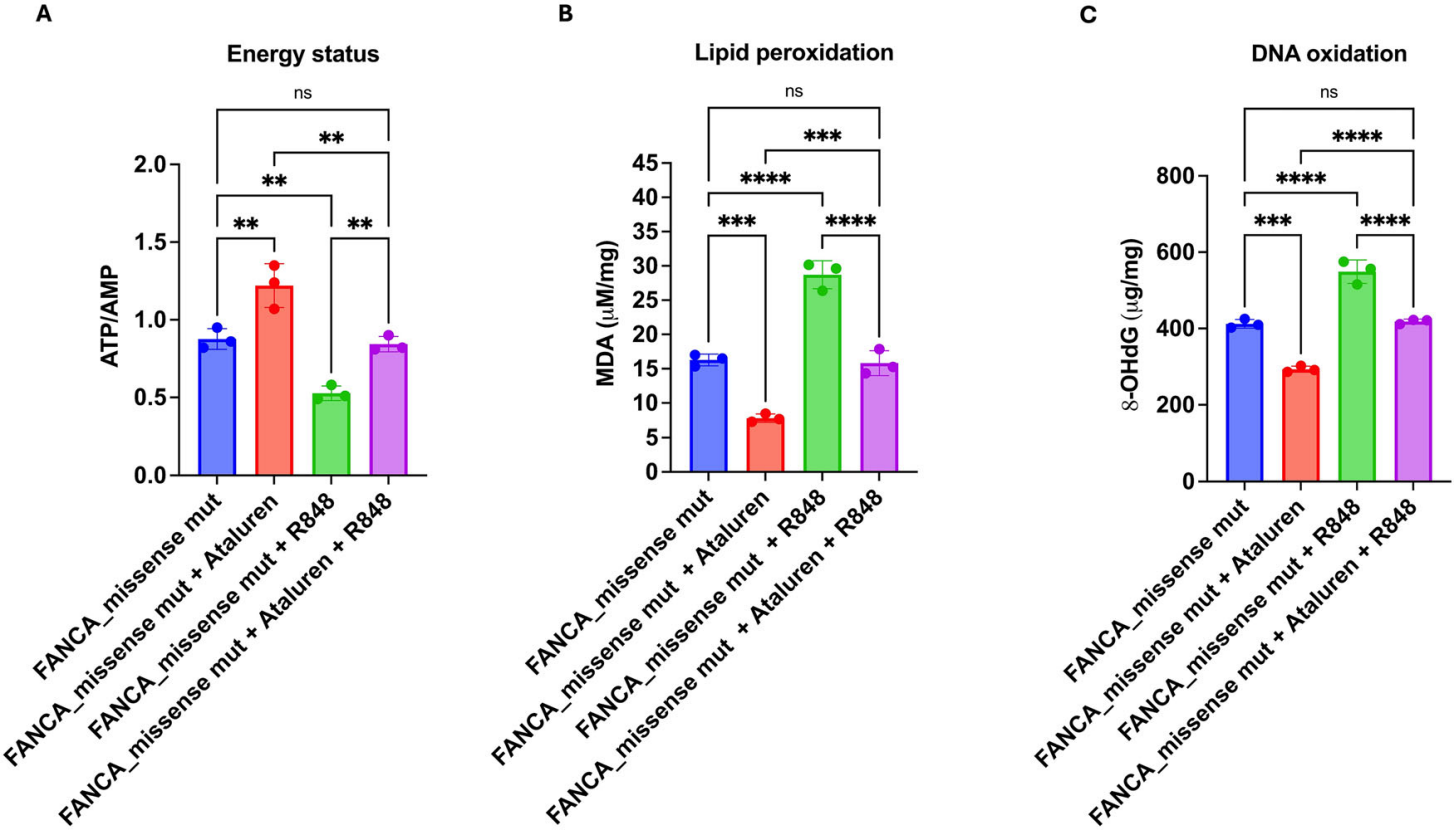
Effect of ataluren treatment on energy status and oxidative damage accumulation in FANCA lymphoblasts previously treated with R848. All analyses were performed on lymphoblasts isolated from FA patients carrying missense mutations (mut). Cells were treated with 10 µM R848 for 24 h prior ataluren treatment (2.5 µM for 72 h) (**A**) ATP/AMP ratio, as a marker of energy status. **B** Malondialdehyde (MDA) intracellular concentration, as a lipid peroxidation marker. **C** 8-hydroxy-2’-deoxyguanosine (8-OHdG) content, as a DNA oxidation marker. Data are presented as mean ± SD and are representative of three independent experiments (*n* = 3). **, ***, and **** indicate statistically significant differences at *p* < 0.01, 0.001, and 0.0001, respectively. ns indicates no statistically significant difference.

Although the metabolic defects of FA cells are already detectable under basal conditions, we sought to determine whether these defects would be exacerbated upon immune stimulation with R848 and whether ataluren could counteract the resulting metabolic stress. Specifically, the R848 treatment resulted in a 39.59 ± 8.08% reduction in the ATP/AMP ratio compared to untreated FA cells. However, co-treatment with ataluren effectively restored cellular energy levels similar to those of untreated FA cells (Fig. 5A), modulating both ATP and AMP concentrations (Supplementary Fig. 1).

Furthermore, the relative percentage increase in the ATP/AMP ratio induced by ataluren was comparable between unstimulated and R848-stimulated conditions (increase in the absence of R848: 38.93 ± 7.38%; in the presence of R848: 61.37 ± 22.02%; *p* = 0.1694). R848 stimulation also led to significant increases in MDA and 8OHdG levels—by 76.38 ± 3.86% and 33.17 ± 8.82%, respectively—indicating enhanced lipid peroxidation and oxidative DNA damage (Fig. 5B, C, respectively). However, ataluren treatment successfully counteracted these effects, reducing MDA and 8OHdG levels to values similar to those of unstimulated FA cells.

Consistent with the ATP/AMP ratio data, ataluren’s protective effect on oxidative damage markers was comparable under basal and R848-stimulated conditions. Specifically, for MDA, the percentage reduction was 52.15 ± 3.20% in the absence of R848 and 44.87 ± 5.93% in its presence (*p* = 0.1344); for 8OHdG, the reduction was 28.84 ± 3.38% without R848 and 23.64 ± 3.30% with R848 (*p* = 0.1294).

### Ataluren-dependent improvement in OxPhos efficiency may be mediated by a recovery of mitochondrial dynamics and turnover, as well as the reduced hyperphosphorylation of mTOR and the S6 axis

**Fig. 6:**
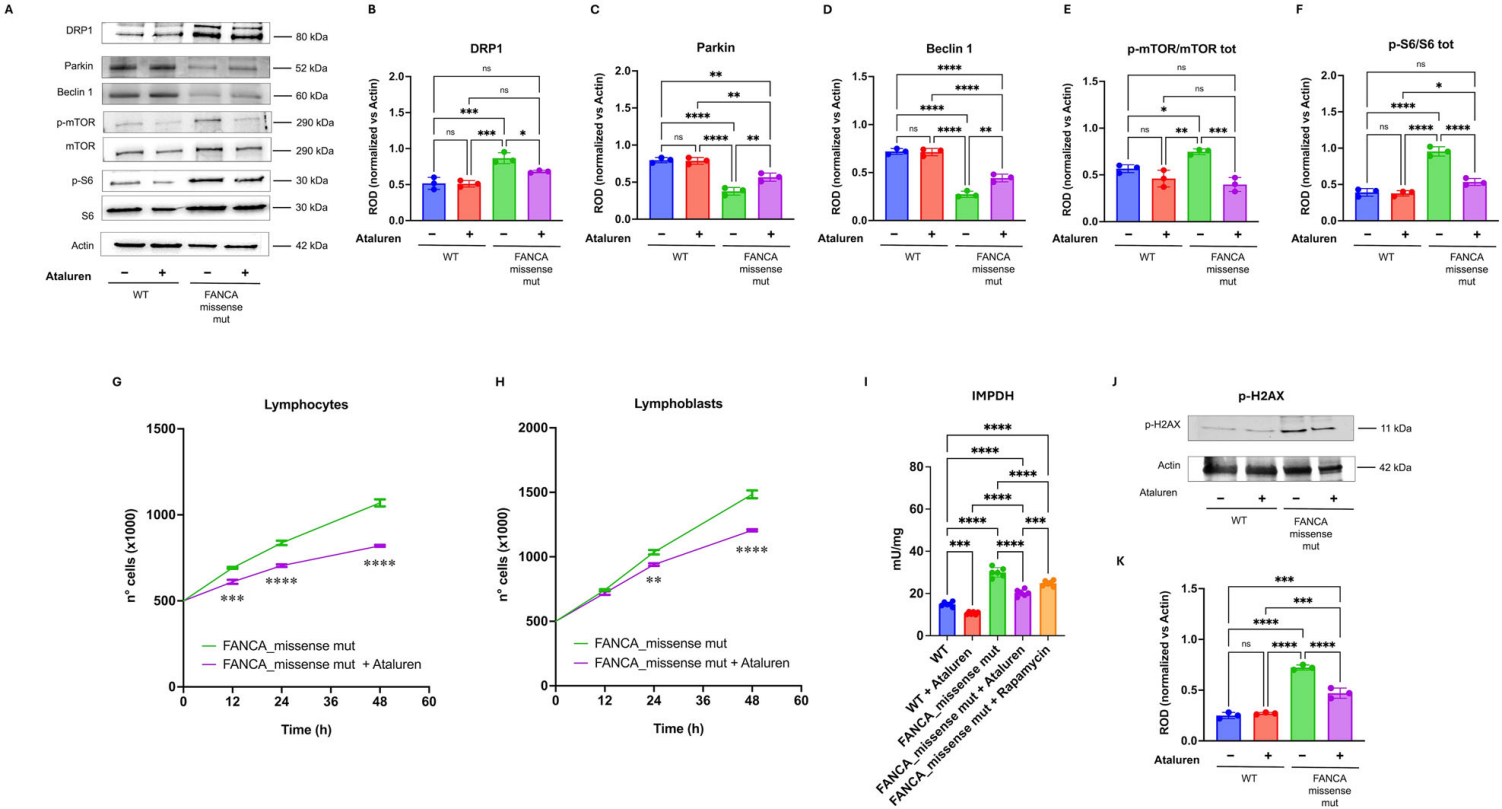
Effect of ataluren treatment on DRP1 expression, mTOR-S6 pathway, cell growth, and DNA damage in FANCA cells. **A** Representative western blot (WB) signals of DRP1, Parkin, Beclin 1, p-mTOR, mTOR, p-S6, and S6 in WT and missense FANCA-mutated lymphoblasts treated with 2.5 µM ataluren for 72 h. Actin was used as a loading control. **B** Densitometric analysis of DRP1 signal. **C** Densitometric analysis of Parkin signal. **D** Densitometric analysis of Beclin 1 signal. **E** Ratio of phosphorylated to total mTOR signal. **F** Ratio of phosphorylated to total S6 signal. **G** Growth of FANCA lymphocytes in the absence (green line) or presence (purple line) of 2.5 µM ataluren. **H** Growth of FANCA lymphoblasts in the absence (green line) or presence (purple line) of 2.5 µM ataluren. **I** IMPDH activity in WT and missense FANCA-mutated lymphoblasts treated with 2.5 µM ataluren for 72 h. Missense FANCA-mutated lymphoblasts were also treated with 1 µM rapamycin for 24 h. **J** Representative WB signals of phospho-H2AX. **K** Densitometric analysis of phospho-H2AX signal. Data are presented as mean ± SD and are representative of three independent experiments (*n* = 3). *, **, ***, and **** indicate statistically significant differences at *p* < 0.05, 0.01, 0.001, and 0.0001, respectively. ns indicates no statistically significant difference.

Given our recent findings highlighting a correlation between mitochondrial dysfunction and hyper-fragmentation of the mitochondrial network driven by increased DRP1 expression [19], we assessed DRP1 protein levels following ataluren treatment (2.5 μM for 72 h). As expected, FA lymphoblasts showed elevated DRP1 levels compared to healthy controls. Notably, ataluren treatment significantly reduced DRP1 expression, restoring it similar to that observed in healthy donor cells (Fig. 6A, B).

Ataluren also exerted a positive effect on mitochondrial turnover, as it caused a recovery in the expression of Parkin, an E3 ubiquitin ligase involved in mitochondrial polyubiquitination [30] (Fig. 6A, C) and Beclin 1, a protein that facilitates the translocation of the Parkin (PARK2) protein from the cytoplasm to the mitochondria [31] (Fig. 6A, D) in FANCA missense-mutated lymphoblasts, although without reaching the levels of healthy control.

In addition, since FANCA-mutated cells are characterized by hyperphosphorylation of mTOR, the effect of ataluren on this protein and on one of its effectors, the S6 protein, was investigated. Ataluren treatment effectively reduced the phosphorylation of both proteins to levels similar to those in healthy controls (Fig. 6A, E, F, respectively).

### Ataluren slows cell proliferation and reduces inosine monophosphate dehydrogenase activity, thereby mitigating DNA damage accumulation in FA cells

To investigate the biological role of ataluren in FA cells, we evaluated the proliferative capacity of primary lymphocytes and lymphoblastoid cell lines carrying a missense mutation in the FANCA gene, given the pivotal role of mTOR in this process [32, 33]. In both models, a significant reduction in proliferation was observed following treatment with 2.5 μM ataluren (Fig. 6, Panels G and H). Notably, 2.5 μM ataluren also reduced cell number in wild-type lymphocytes and lymphoblastoid cells, although to a lesser extent (Supplementary Fig. 2). In addition, both for FANCA and WT cells, the effect on the proliferation rate was dose-dependent, as a further reduction in cell number was observed after treatment with 5 or 10 μM ataluren (Supplementary Fig. 3).

Lymphoblasts harboring the *FANCA* missense mutation also exhibited a decrease in inosine monophosphate dehydrogenase (IMPDH) activity after treatment (Fig. 6I). Notably, a similar reduction in IMPDH activity was observed following treatment with rapamycin, the specific inhibitor of mTOR signaling pathway (Fig. 6I).

Overall, the observed cell cycle slowdown, together with decreased levels of 8OHdG (Fig. 4B), appears to contribute positively to limiting DNA damage accumulation, as evidenced by reduced p-H2AX expression in FA lymphoblasts treated with ataluren (Fig. 6J, K).

## Discussion

For many years, ataluren has been evaluated in various clinical trials for its ability to promote the readthrough of nonsense mutations in patients with genetic disorders [8]. For example, it was recently tested in FA cell lines carrying stop codon mutations, exploiting its readthrough potential [28]. These studies demonstrated increased expression of FA genes, which in turn enhanced the DNA damage response and modulated p53 protein levels, thereby reducing the risk of malignant transformation [28].

Since FA cells are characterized not only by defective DNA repair but also by impaired energy metabolism [18], we investigated whether ataluren could restore mitochondrial function in homozygous *FANCA* nonsense mutant cells, potentially as a consequence of partial restoration of FANCA protein expression.

Our data show that ataluren modulates mitochondrial metabolism and cellular energy status in a dose- and time-dependent manner. However, the most compelling finding is that ataluren not only affects OxPhos activity and efficiency in *FANCA* nonsense mutant lymphoblasts through the improvement of cellular energy status and the consequent decrease in oxidative damage but also exerts similar effects in lymphoblasts carrying *FANCA* missense mutations. These results suggest that ataluren influences mitochondrial metabolism regardless of its readthrough activity. Notably, ataluren also has a detectable metabolic effect in WT lymphoblasts, albeit to a lesser extent, further supporting a direct role in modulating cellular energy metabolism beyond its action on nonsense mutations. More specifically, ataluren impairs cellular energy metabolism by slowing both aerobic ATP synthesis and OCR in the presence of either respiratory substrate (P/M and Succ). Interestingly, this apparently negative effect on OxPhos metabolism leads to an improvement in the coupling efficiency between respiration and ATP production when cells are energized with P/M. Specifically, the P/O ratio in *FANCA*-mutated cells is restored to levels comparable to those of healthy controls, whereas the ratio measured in the presence of Succ remains unchanged—likely because it was already similar to control values at baseline. In addition, despite the decrease in aerobic metabolism, neither *FANCA*-mutated nor control cells appear to compensate by increasing anaerobic glycolysis, as indicated by unchanged lactate release, anaerobic glycolysis yield, and LDH activity. At the lowest tested concentration (2.5 μM), ataluren improves the cellular energy status in cells carrying either nonsense or missense FANCA mutations. This improvement appears to result primarily from reduced AMP levels rather than increased intracellular ATP content, which remains stable. In contrast, higher concentrations of ataluren (5 and 10 μM) lead to either no effect or a decline in energy status, likely due to further suppression of OxPhos activity and a corresponding drop in ATP levels. This raises a critical question: how can ataluren at 2.5 μM reduce glucose consumption and aerobic ATP synthesis without affecting total intracellular ATP levels while lowering intracellular AMP levels? One possible explanation lies in the improved efficiency of OxPhos. Enhanced coupling between oxygen consumption and ATP synthesis reduces ROS production [34, 35] and the consequent oxidative damage accumulation, as shown by the reduction of 8OHdG and MDA intracellular levels, thereby preventing a pro-oxidant vicious cycle that could compromise mitochondrial integrity, particularly the inner membrane [36]. In other words, increased OxPhos efficiency lowers the energy demand required for antioxidant defenses and repair pathways that counteract oxidative stress.

The beneficial effects of ataluren on cellular energy status and oxidative damage are also evident when *FANCA*-mutated cells are stimulated with R848. While R848 alone decreases the ATP/AMP ratio and increases levels of MDA and 8OHdG, co-treatment with ataluren attenuates these effects, restoring energy balance and reducing oxidative markers to levels comparable to untreated cells. Given that infections in FA patients exacerbate bone marrow failure [37, 38]—partly due to the high turnover required by circulating lymphocytes with metabolic and DNA repair deficiencies—ataluren may improve the survival of activated lymphocytes by preserving energy homeostasis and limiting oxidative stress.

Besides restoring FANCA protein synthesis, a possible mechanism for ataluren’s direct effect on cellular energy status recovery may involve decreased hyperphosphorylation of mTOR and the slowdown of the cellular proliferation rate. In detail, ataluren treatment reduces phosphorylated mTOR (p-mTOR) levels without affecting total mTOR levels, indicating a modulatory effect on mTOR signaling rather than on its expression. Since mTOR is a serine/threonine kinase that plays a pivotal role in regulating energy metabolism, mitochondrial function, autophagy, and cell proliferation [32, 33], its modulation mediated by ataluren could impact energy metabolism in *FANCA*-mutant cells, acting directly on mitochondrial function, as well as cause a reduction in cell proliferation, leading to a saving in energy expenditure. Additionally, ataluren reduces the phosphorylation of ribosomal protein S6, a downstream mTOR target, which is also involved in cell growth and glucose metabolism [39]. From a biological perspective, the slowdown of cell proliferation may be beneficial not only for the energy balance but also for the DNA repair process. By delaying cell cycle progression, cells may activate alternative pathways to resolve DSBs, as suggested by reduced phosphorylation of H2AX, a marker of DSBs, in ataluren-treated *FANCA*-mutated cells. However, it is important to note that the complete mTOR pathway switch-off obtained with rapamycin treatment results in effects different from those observed with ataluren. A study published by our group in Metabolites [40] reports that rapamycin treatment caused a reduction in oxidative damage but also a nearly complete loss of OxPhos functionality, without improving its efficiency. This drastic suppression of oxidative metabolism forced the cells to increase anaerobic glycolysis, probably to compensate for the energy deficit caused by OxPhos shutdown, which ultimately resulted in an even lower ATP/AMP ratio. Therefore, it is possible to speculate that while mTOR modulation may play a key role in the recovery of aerobic metabolism in FA cells, excessive inhibition of this pathway could be detrimental.

Another possible explanation for the effects of ataluren on energy metabolism is the reduction in expression levels of DRP1, a key regulator of mitochondrial fission, overexpressed in *FANCA*-mutated cells compared to healthy controls [19]. It is well established that the balance between mitochondrial fission and fusion determines mitochondrial dynamics, function, and efficiency [41], and that excessive fission can disrupt the mitochondrial network and increase the risk of OxPhos uncoupling and consequent ROS production [42]. Thus, a reduction of DRP1 levels in cells harboring *FANCA* missense mutations induced by ataluren treatment could contribute to the improvement of ATP/OCR coupling observed under P/M stimulation. Interestingly, the effect on DRP1 expression could be related to the modulation of mTOR signaling, as literature reports that mTORC1 regulates the mitochondrial dynamics through the recruitment of DRP1 via 4E-BPs [43].

In addition, ataluren partially restores the expression levels of Parkin and Beclin 1, two proteins involved in the process of mitophagy [30, 31], which in FANCA cell lines appear to be lower expressed compared to healthy controls [19]. Considering that mitophagy is essential for proper mitochondrial turnover and prevents the accumulation of damaged mitochondria and the associated ROS production [44], its recovery may contribute to the reduction of oxidative damage accumulation and the improvement of aerobic metabolism.

Taking all these factors into account, it could be speculated that the improvement in the cellular energy state observed upon treatment with 2.5 µM ataluren likely results from the establishment of a new metabolic balance—characterized by enhanced OxPhos efficiency and reduced energy expenditure to counteract oxidative damage, probably due to a slowing of the proliferation rate associated with the modulation of mTOR/S6 axis and improved mitochondrial quality control and dynamics.

*FANCA*-mutated cells also display elevated IMPDH activity compared to controls, which is significantly reduced by ataluren treatment, although not to WT levels. In this case as well, the ataluren effect on IMPDH activity could depend on the modulation of mTOR signaling, as mTOR controls de novo purine synthesis through regulation of the mitochondrial tetrahydrofolate cycle [45]. Supporting this hypothesis, rapamycin (the specific mTOR inhibitor) also reduces IMPDH activity. However, as already mentioned above, ataluren and rapamycin do not produce identical effects in FA cells, suggesting that ataluren may act on IMPDH through additional or alternative mechanisms. Therefore, a second hypothesis could be proposed: ataluren primarily targets mitochondrial metabolism, with changes in IMPDH activity as a downstream consequence. In fact, it is known that impaired mitochondrial respiration inhibits the final steps of de novo purine biosynthesis, leading to accumulation of IMP possibly in response to elevated AMP levels [46].

In conclusion, although several mechanistic aspects of ataluren’s effects on cellular metabolism remain to be elucidated, our findings strongly support a dual role for this drug: promoting readthrough of nonsense mutations and modulating mitochondrial and metabolic functions. Together, these actions may contribute to improved cellular homeostasis in FA and potentially in other IBMFS.

## Materials and methods

### Samples

FA lymphoblast cell lines that carried out nonsense or missense mutations of the *FANCA* gene were obtained from the “Cell Line and DNA Biobank from Patients affected by Genetic Diseases” (IRCCS Istituto Giannina Gaslini, Genoa, Italy)—Telethon Genetic Biobank Network (Project No. GTB07001). In detail, two cell lines derived from two patients with *FANCA* nonsense mutations and three cell lines derived from three patients with *FANCA* missense mutations were employed. Primary lymphocytes were isolated using Ficoll-Hipaque Plus (GE Healthcare Biosciences, Piscataway, NJ) from three patients with *FANCA* missense mutations. As controls, two lymphoblast cell lines and lymphocytes from three healthy donors were used. All cell lines were tested for the absence of mycoplasma contamination.

The study was conducted following the Declaration of Helsinki and approved by the regional ethics committee, protocol JS002, register number 037−21/01/2019. All the subjects or their legal guardians gave written informed consent to the investigation.

### Cell culture conditions

Lymphoblast cell lines and primary lymphocytes were grown at 37 °C with a 5% CO_2_ in RPMI-1640 medium (GIBCO, Billing, MT, USA) containing 10% fetal bovine serum (FBS, Euroclone, Milano, Italy), 100 U/mL penicillin, and 100 µg/mL streptomycin (Euroclone, Milano, Italy). Cells were counted using a Burker chamber after trypan blue staining to evaluate the cellular growth rate.

Cells were treated for 24, 48, and 72 h with 2.5, 5, or 10 µM ataluren (Selleck Chemicals, Houston, TX, USA). In some cases, cells were treated with 10 µM R848 (Resiquimod; Merck, Darmstadt, Germany), a Toll-like receptor agonist that mimics bacterial infection [47], for 24 h before ataluren treatment. In other cases, cells were treated with 1 µM rapamycin for 24 h to inhibit mTOR signaling.

### ATP and AMP intracellular concentration assay and evaluation of cellular energy status

ATP and AMP levels were quantified using 50 µg of total protein.

ATP determination was carried out spectrophotometrically with a double-beam spectrophotometer (UNICAM UV2, Analytical S.n.c., Borgotaro, PR, Italy) by monitoring NADP reduction at 340 nm. The reaction mixture contained 100 mM Tris-HCl (pH 8.0; Merck, Darmstadt, Germany), 0.2 mM NADP, 5 mM MgCl₂, 50 mM glucose, and 3 µg of exogenous enzymes (hexokinase and glucose-6-phosphate dehydrogenase; Merck, Darmstadt, Germany) [48].

AMP was measured spectrophotometrically by following NADH oxidation at 340 nm. The assay buffer consisted of 100 mM Tris-HCl (pH 8.0; Merck, Darmstadt, Germany), 5 mM MgCl₂, 0.2 mM ATP, 10 mM phosphoenolpyruvate, 0.15 mM NADH, and the auxiliary enzymes adenylate kinase (10 IU), pyruvate kinase (25 IU), and lactate dehydrogenase (15 IU) (all from Merck, Darmstadt, Germany) [48].

Cellular energy status was expressed as the ATP/AMP ratio, calculated from the respective intracellular concentrations.

### OxPhos function and efficiency assay

OxPhos function was evaluated by measuring OCR and FoF₁-ATP synthase activity.

OCR was determined using an amperometric oxygen electrode (Unisense Microrespiration, Aarhus, Denmark) in a sealed chamber. For each measurement, 1 × 10⁵ cells were permeabilized with 0.03 mg/ml digitonin for 1 min prior to analysis. To stimulate respiration through Complex I or Complex II, cells were supplied with 10 mM pyruvate plus 5 mM malate or with 20 mM succinate, respectively (all reagents from Merck, Darmstadt, Germany) [49].

FoF₁-ATP synthase activity was assessed in 1 × 10⁵ cells suspended in PBS supplemented with 0.6 mM ouabain (Na⁺/K⁺-ATPase inhibitor) and 0.25 mM diadenosine-5′-pentaphosphate (adenylate kinase inhibitor; Merck, Darmstadt, Germany). After 10 min incubation, respiration was stimulated with either 10 mM pyruvate plus 5 mM malate or 20 mM succinate. ATP generation was quantified using a luminometer (GloMax® 20/20, Promega Italia, Milan, Italy) and a luciferin/luciferase-based bioluminescence assay kit (CLS II, #11699695001; Roche, Basel, Switzerland). Luminescence was recorded every 30 s for a total of 2 min [49].

OxPhos efficiency was expressed as the P/O ratio, defined as the number of ATP molecules synthesized per atom of oxygen consumed. Under physiological conditions, mitochondria typically show P/O ratios of ~2.5 with P/M and ~1.5 with Succ [50]. Values below these benchmarks indicate impaired coupling between oxygen consumption and ATP synthesis, suggesting inefficient oxidative metabolism and potentially enhanced ROS generation [51].

### LDH activity assay

LDH (EC 1.1.1.27) activity was determined spectrophotometrically by monitoring NADH oxidation at 340 nm. The assay solution contained 100 mM Tris-HCl buffer (pH 7.4), 0.2 mM NADH, and 5 mM pyruvate [52].

### Glucose consumption and lactate release evaluation

Glucose consumption was assessed by monitoring NADP⁺ reduction at 340 nm. 5 µL of cell culture medium was mixed with a reaction buffer containing 50 mM Tris-HCl (pH 8.0), 1 mM NADP⁺, 10 mM MgCl₂, and 2 mM ATP. The absorbance was measured spectrophotometrically before and after the addition of 4 µg of purified hexokinase (HK) and glucose-6-phosphate dehydrogenase (G6PD), as previously described [53].

Lactate content released into the medium was determined by following the reduction of NAD⁺ at 340 nm. In this assay, 10 µL of culture medium was added to a solution containing 100 mM Tris-HCl (pH 8.0) and 5 mM NAD⁺. Absorbance was recorded before and after the addition of 4 µg of purified lactate dehydrogenase [53].

All measurements were normalized to the number of cells.

### Oxidative damage markers evaluation

Lipid peroxidation was assessed using the thiobarbituric acid reactive substances (TBARS) assay, which quantifies MDA as an end-product of lipid oxidative degradation. The TBARS reagent consisted of 0.25 M HCl, 0.25 mM trichloroacetic acid, and 26 mM thiobarbituric acid (all from Merck, Darmstadt, Germany). For each assay, 50 µg of protein diluted in 300 µl of Milli-Q water was mixed with 600 µl of TBARS reagent. Samples were incubated at 95 °C for 1 h to allow MDA-thiobarbituric acid adduct formation, and absorbance was subsequently recorded at 532 nm. MDA standards (1–20 µM) were used to generate a calibration curve for quantification [53].

Oxidative DNA damage was evaluated by measuring 8-OHdG levels using a commercially available ELISA kit (#ab201734, Abcam, Cambridge, UK), following the manufacturer’s protocol.

### Western blot analysis

Western blot (WB) analysis was carried out by denaturing gel electrophoresis (SDS-PAGE) using 30 µg of protein per lane, separated on 4–20% gradient gels (BioRad, Hercules, CA, USA). Proteins were transferred onto membranes and probed with the following primary antibodies: DRP1 (#8570S, Cell Signaling Technology, Beverly, MA, USA), anti-Parkin (#PA5-13399 ThermoFisher, Waltham, MA, USA), anti-Beclin1 (#3495 P, Cell Signaling Technology, USA), phospho-mTOR (Ser2448; #5536S, Cell Signaling Technology), mTOR (#2983S, Cell Signaling Technology), phospho-S6 ribosomal protein (Ser235/236; #2211S, Cell Signaling Technology), S6 ribosomal protein (#2217S, Cell Signaling Technology), phospho-H2AX (#05–636, Merck, Darmstadt, Germany), and β-actin (#MA1-140, ThermoFisher Scientific, Waltham, MA, USA). Primary antibodies were diluted in PBS containing 0.15% Tween-20 (PBSt; Roche, Basel, Switzerland), following the manufacturer’s recommendations.

Appropriate HRP-conjugated secondary antibodies (Merck, Darmstadt, Germany) were used at 1: 10,000 dilution in PBSt. Immunoreactive bands were visualized using an enhanced chemiluminescence substrate (ECL, BioRad, Hercules, CA, USA) and detected with a chemiluminescence imaging system (Alliance 6.7 WL 20 M, UVITEC, Cambridge, UK). Band intensity was quantified using UV1D software (UVITEC, Cambridge, UK). To account for variations in protein loading, all target signals were normalized to β-actin detected on the same membrane.

### IMPDH activity assay

IMPDH (EC:1.1.1.205) was measured following the NAD^+^ reduction at 340 nm. The assay solution contained 100 mM Tris-HCl buffer (pH 7.4), 1 mM NAD^+^, and 0.5 mM inosine monophosphate [52].

### Statistical analysis

All experiments were performed in three independent biological replicates. Given the technical nature of the assays and the low variability observed in previous studies [19, 20, 23, 24, 40], triplicates were considered adequate to detect the expected effect size with acceptable precision. Data are presented as mean ± standard deviation (SD). Statistical comparisons among groups were conducted using one-way analysis of variance (ANOVA), followed by Tukey’s post hoc multiple comparison test, implemented in Prism 9 software (GraphPad Software Inc., Boston, MA, USA). A *p*-value < 0.05 was considered statistically significant. Significance levels were annotated as follows: ns = not significant; * *p* < 0.05; ** *p* < 0.01; *** *p* < 0.001; **** *p* < 0.0001.

## Supplementary information

Supplementary Materials

Western blot original signals

Supplementary Table I

## Data Availability

All data generated or analyzed in this study are included in the published article and its supplementary information files.
