# Supplementary material for: Beyond readthrough: ataluren restores mitochondrial function and reduces oxidative stress in FANCA-mutated cells via mTOR–DRP1 modulation
Source: Cell Death Discov. 2026 Feb 28;12:124. doi: 10.1038/s41420-026-02983-6 (PMC13031327; doi:10.1038/s41420-026-02983-6)
Supplement: Supplementary file 1 — Supplementary Materials [file 41420_2026_2983_MOESM1_ESM.pdf]

Supplementary Figure 1. Effect of ataluren treatment on intracellular content of ATP and AMP FANCA lymphoblasts prior treated with R848.

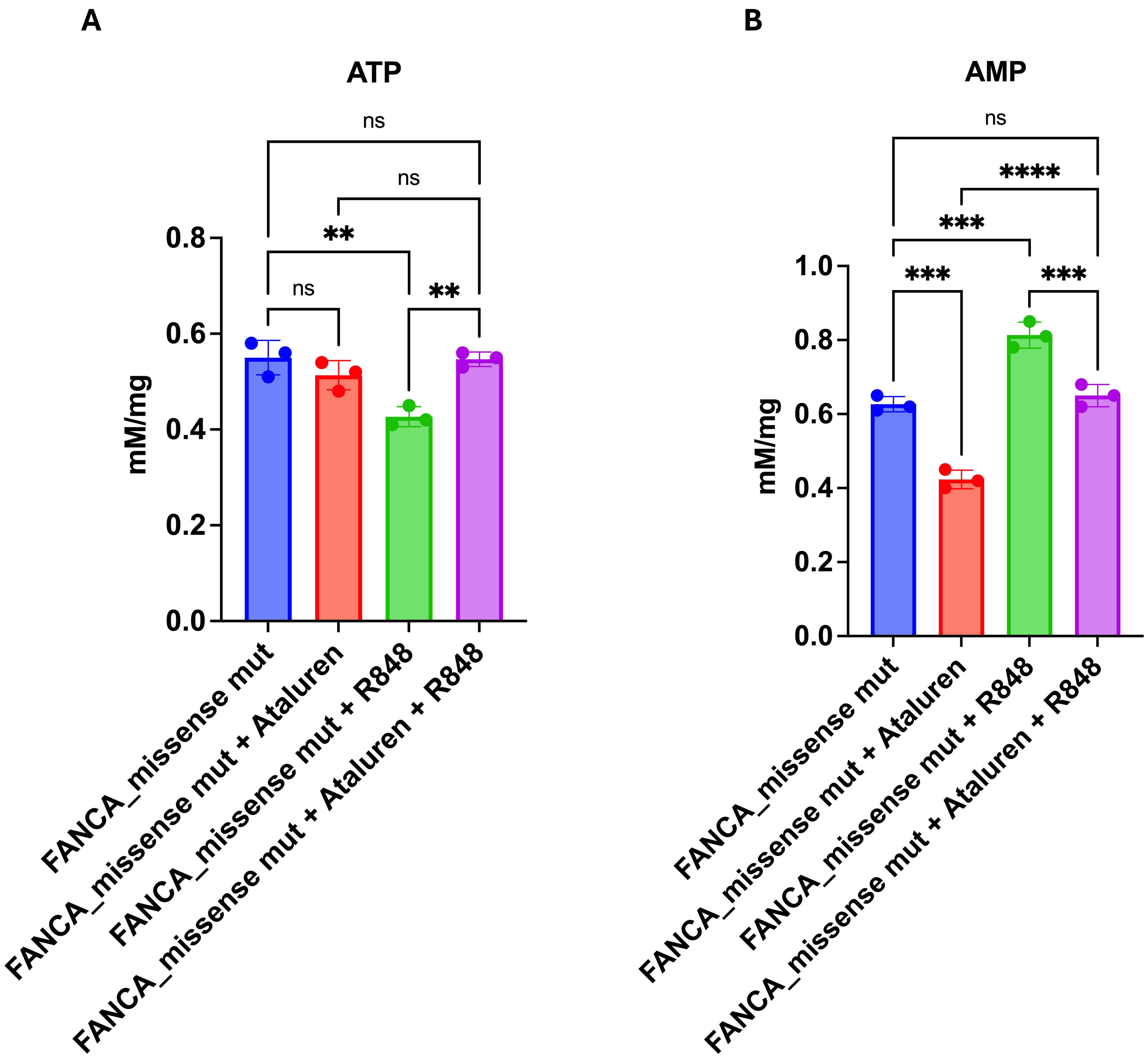

All analyses were performed on lymphoblasts isolated from FA patients carrying missense mutations (mut). Cells were treated with 10 μM R848 for 24 h prior ataluren treatment (2.5 μM for 72h) (A) ATP intracellular content (B) AMP intracellular content.

Data are presented as mean ± SD and are representative of three independent experiments (n = 3). **, ***, and **** indicate statistically significant differences at $p < 0.01$, 0.001, and 0.0001, respectively. ns indicates no statistically significant difference.

Supplementary Figure 2. Effect of ataluren treatment on cell growth in WT lymphocytes and lymphoblasts

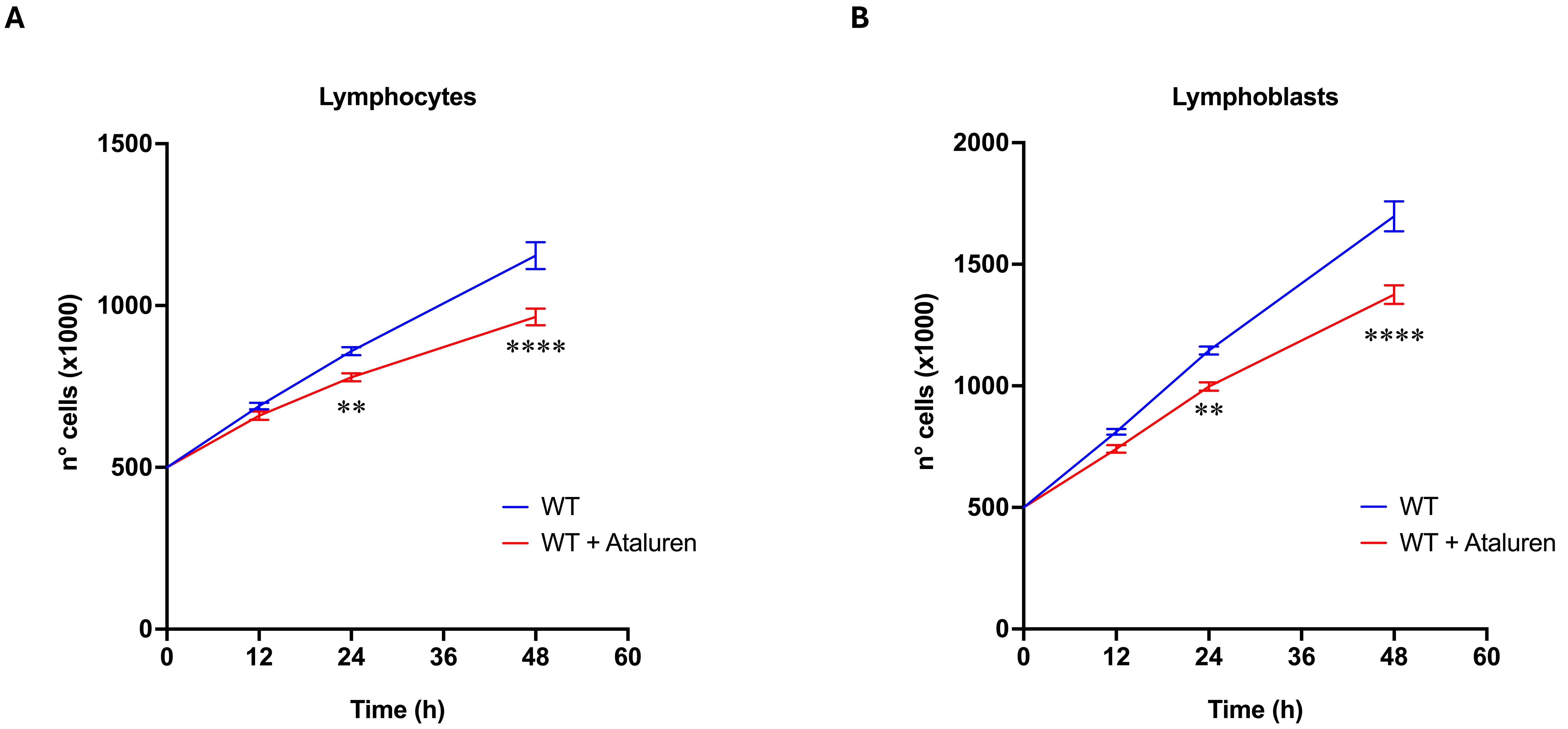

(A) Growth of WT lymphocytes in the absence (blue line) or presence (red line) of 2.5 μM ataluren. (B) Growth of WT lymphoblasts in the absence (blue line) or presence (red line) of 2.5 μM ataluren.

Data are presented as mean ± SD and are representative of three independent experiments (n = 3). ** and **** indicate statistically significant differences at $p < 0.01$ and 0.0001, respectively.

Supplementary Figure 3. Effect of 5 and 10 μM ataluren treatment on cell growth in WT and FANCA lymphoblasts

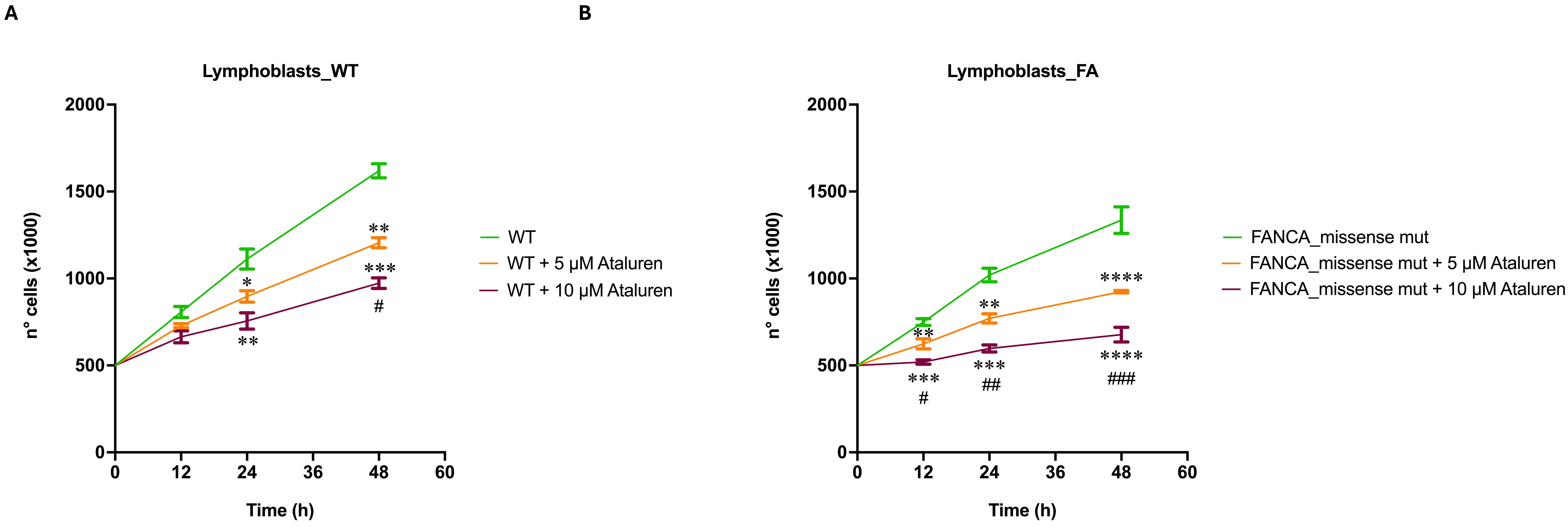

(A) Growth curve of WT lymphoblasts in the absence (green line) or presence of 5 μM (orange line) or 10 μM (violet line) ataluren. (B) Growth of FANCA lymphoblasts in the absence (green line) or presence of 5 μM (orange line) or 10 μM (violet line) ataluren. Data are presented as mean ± SD and are representative of three independent experiments (n = 3). *, **, *** and **** indicate statistically significant differences at $p < 0.05$, 0.01, 0.001 and 0.0001, respectively, between untreated and treated samples. #, ##, and ### indicate statistically significant differences at $p < 0.05$, 0.01, and 0.001, respectively, between sample treated with 5 or 10 μM ataluren.
