# Supplementary material for: Beyond readthrough: ataluren restores mitochondrial function and reduces oxidative stress in FANCA-mutated cells via mTOR–DRP1 modulation
Source: Cell Death Discov. 2026 Feb 28;12:124. doi: 10.1038/s41420-026-02983-6 (PMC13031327; doi:10.1038/s41420-026-02983-6)

Figure 6_Panel A

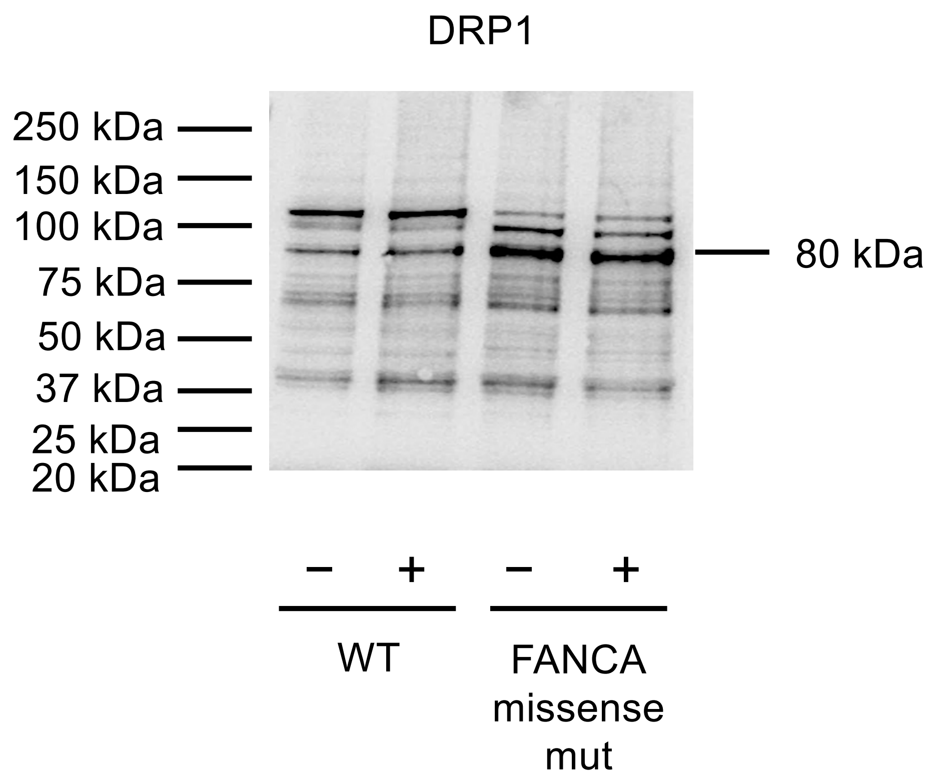

Figure 6_Panel A

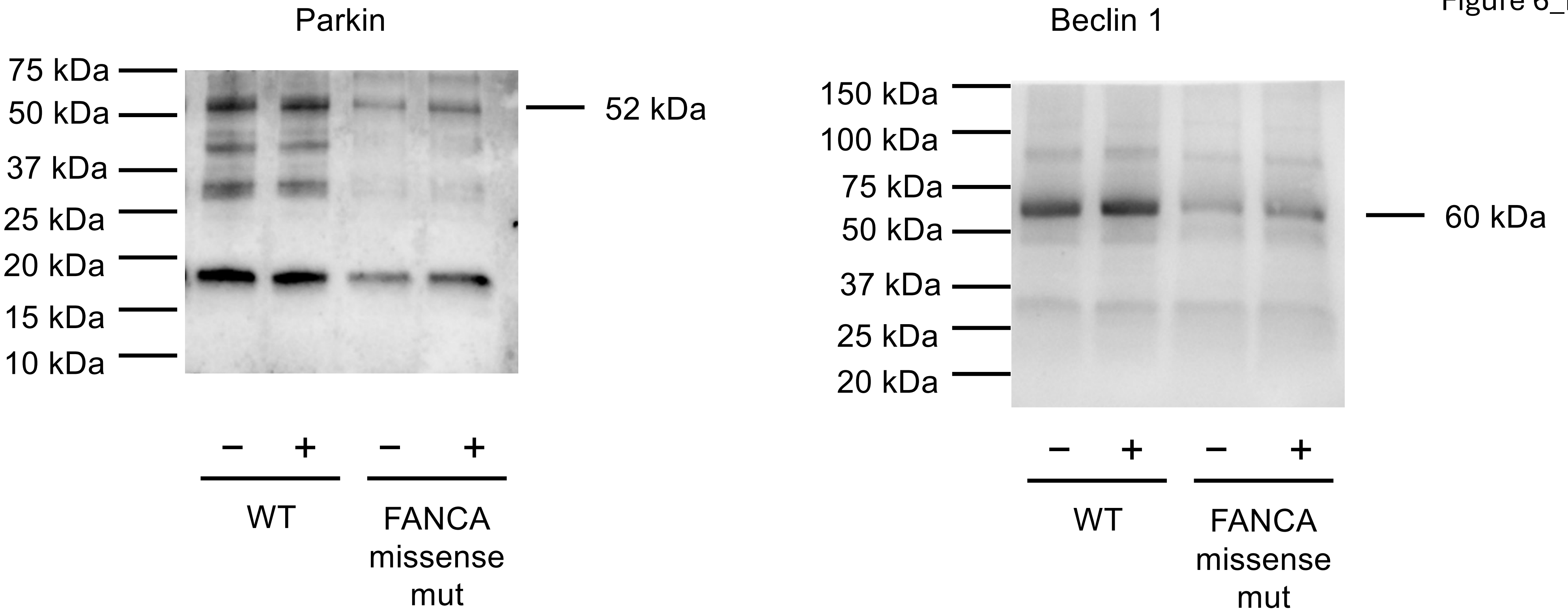

Figure 6_Panel A

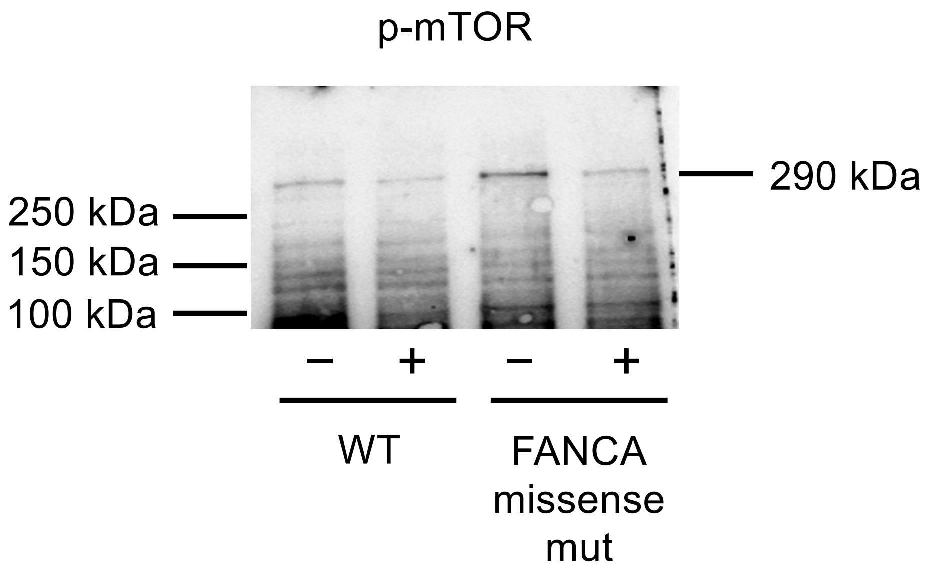

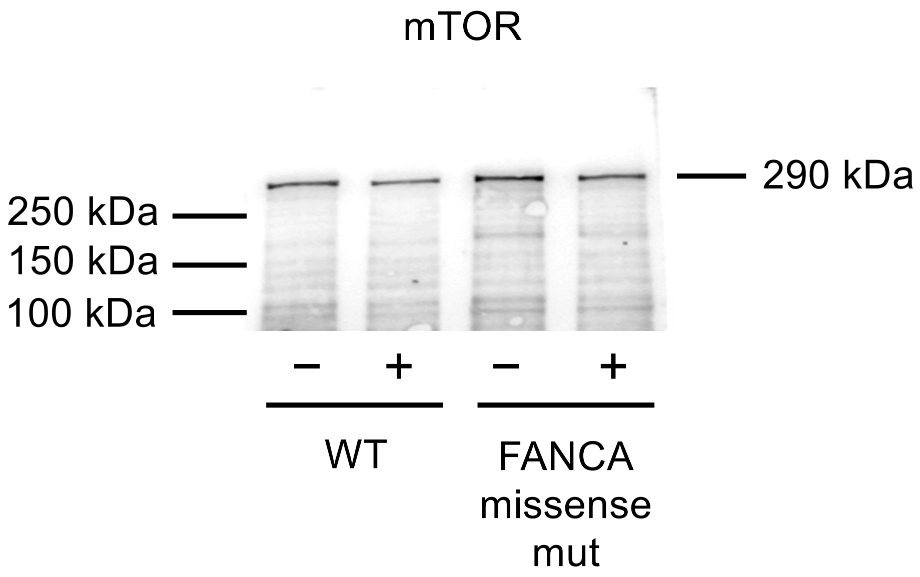

Figure 6_Panel A

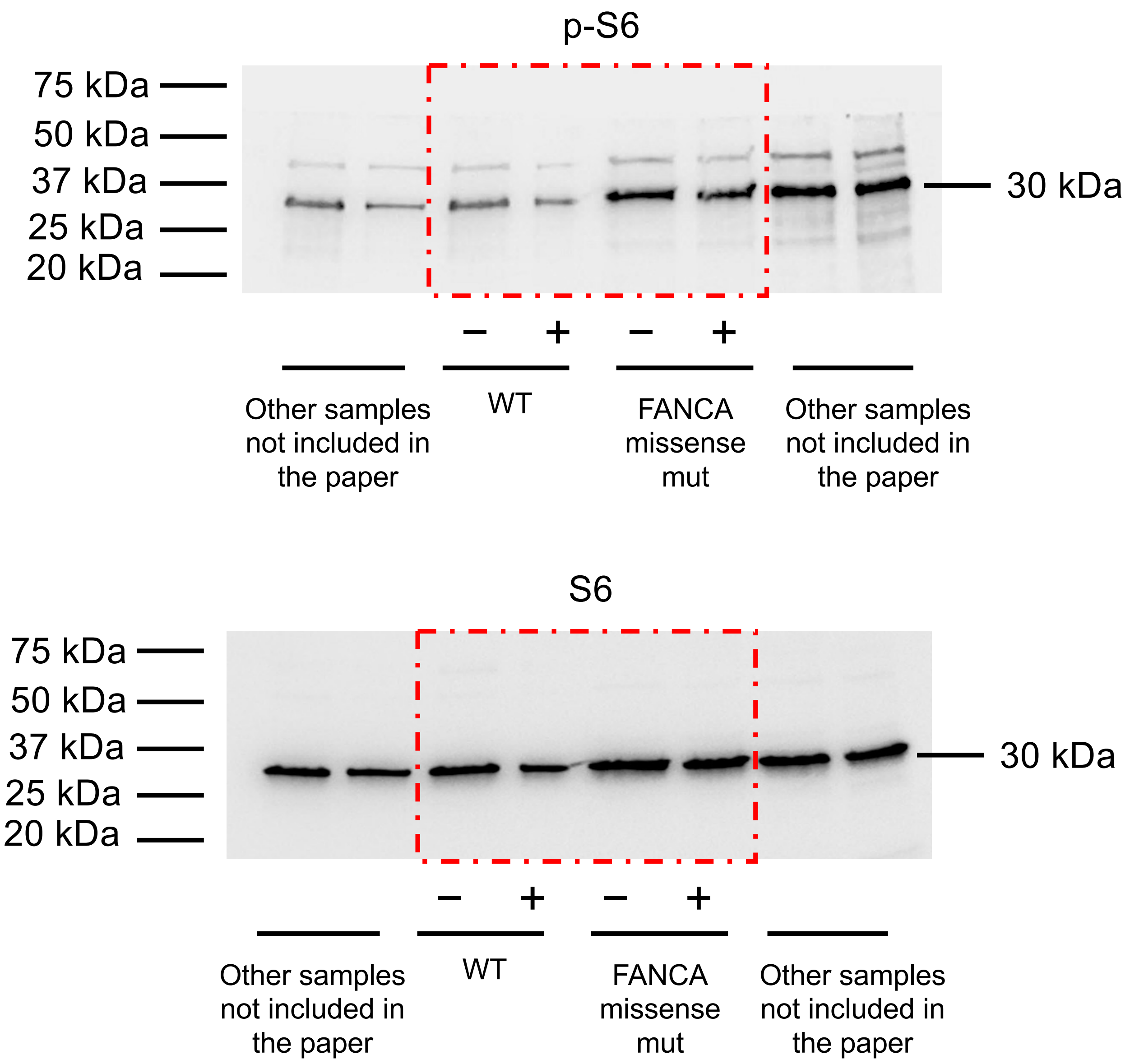

Figure 6_Panel A

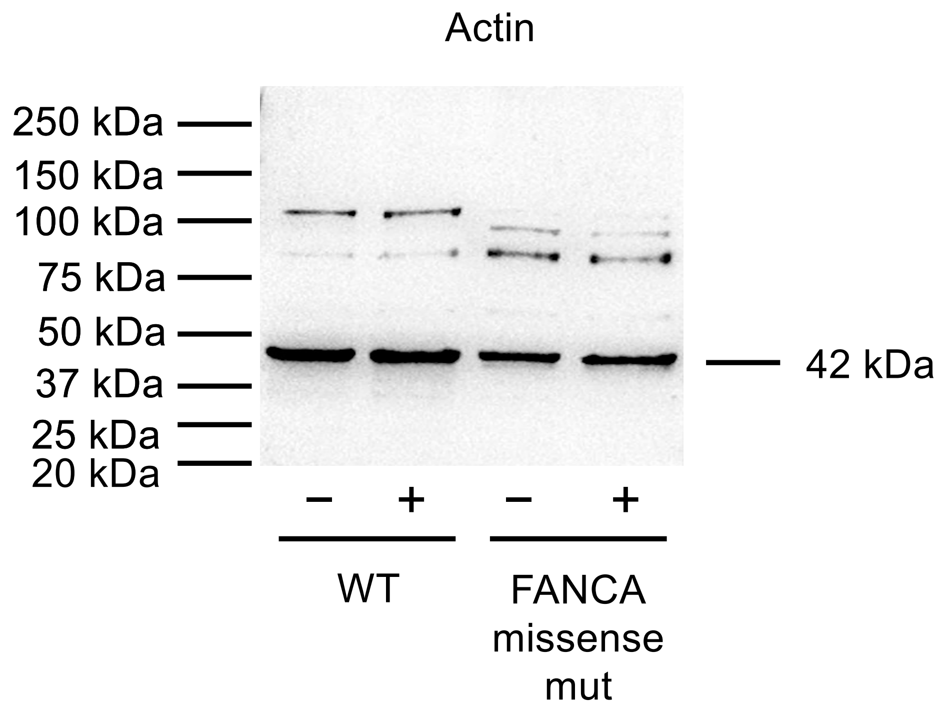

Figure 6_Panel H

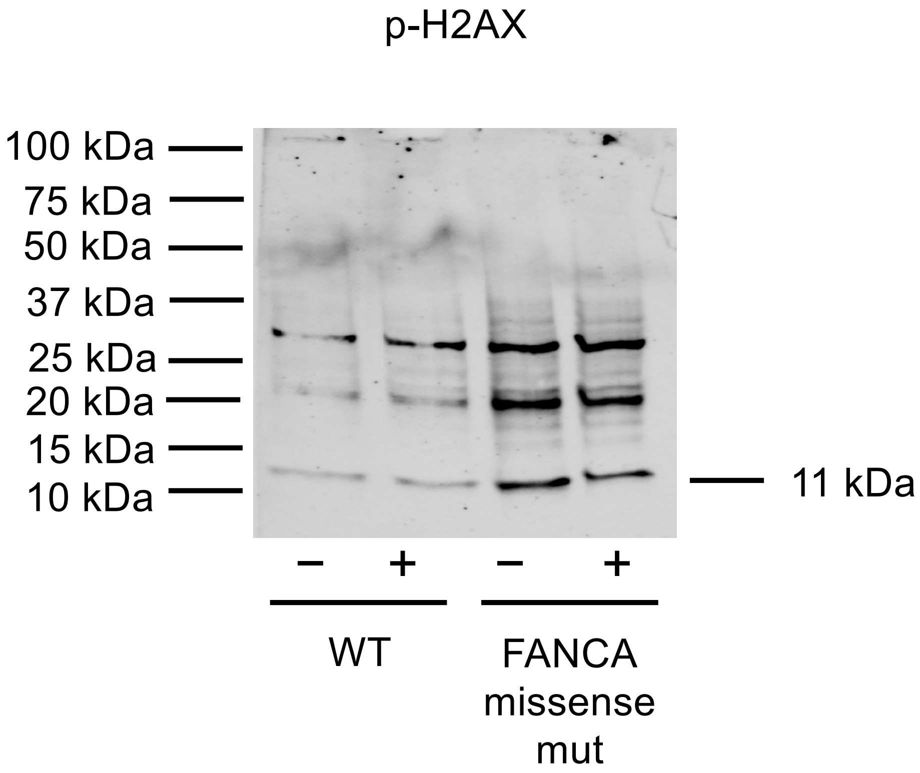

Figure 6_Panel H

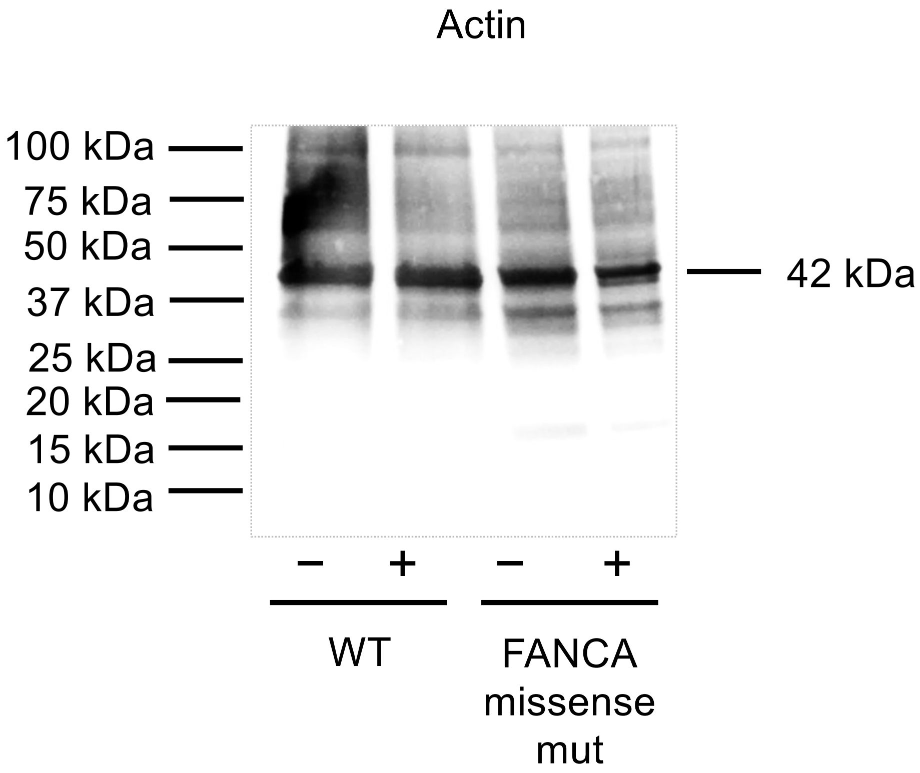

Supplement: Supplementary file 2 — Western blot original signals [file 41420_2026_2983_MOESM2_ESM.pdf]
