# Supplementary material for: Beyond readthrough: ataluren restores mitochondrial function and reduces oxidative stress in FANCA-mutated cells via mTOR–DRP1 modulation
Source: Cell Death Discov. 2026 Feb 28;12:124. doi: 10.1038/s41420-026-02983-6 (PMC13031327; doi:10.1038/s41420-026-02983-6)
Supplement: Supplementary file 3 — Supplementary Table I [file 41420_2026_2983_MOESM3_ESM.docx]

|  | ATP synthesis_P/M | | | OCR_P/M | | | P/O_P/M | | |  |
| --- | --- | --- | --- | --- | --- | --- | --- | --- | --- | --- |
|  | | **2.5 μM_24h** | **10 μM_72h** | **p-value** | **2.5 μM_24h** | **10 μM_72h** | **p-value** | **2.5 μM_24h** | **10 μM_72h** | **p-value** |
| **WT** | | 15.3 ± 1.3 | 60.3 ± 1.7 | <0.0001 | 15.6 ± 2.1 | 59.8 ± 0.9 | <0.0001 | 100,4 ± 0,8 | 98.8 ± 2.1 | 0.2700 |
| **FANCA nonsense mut** | | 53.5 ± 0.7 | 90.3 ± 0.4 | <0.0001 | 55.0 ± 0.1 | 92.6 ± 0.5 | <0.0001 | 103.3 ± 1.6 | 131.8 ± 1.9 | <0.0001 |
| **FANCA missense mut** | | 54.2 ± 0.8 | 90.3 ± 0.7 | <0.0001 | 56.8 ± 0.9 | 92.7 ± 0.5 | <0.0001 | 106.1 ± 2.0 | 131.4 ± 2.4 | <0.0001 |
|  | | **ATP synthesis_Succ** | | | **OCR_Succ** | | | **P/O_Succ** | | |
|  | | **2.5 μM_24h** | **10 μM_72h** | **p-value** | **2.5 μM_24h** | **10 μM_72h** | **p-value** | **2.5 μM_24h** | **10 μM_72h** | **p-value** |
| **WT** | | 13.9 ± 1.4 | 48.3 ± 0.9 | <0.0001 | 10.4 ± 0.6 | 48.7 ± 0.6 | <0.0001 | 97.9 ± 3.1 | 100.7 ± 2.9 | 0.3049 |
| **FANCA nonsense mut** | | 37.8 ± 3.8 | 81.6 ± 0.3 | <0.0001 | 38.5 ± 3.8 | 82.44 ± 0.6 | <0.0001 | 101.2 ± 0.8 | 102.5 ± 2.5 | 0.4320 |
| **FANCA missense mut** | | 36.4 ± 3.7 | 82.1 ± 0.3 | <0.0001 | 37.2 ± 3.9 | 82.3 ± 0.7 | <0.0001 | 100.1 ± 1.5 | 101.6 ± 2.9 | 0.4548 |

**Table I: Comparison between the percentage values of ATP synthase and OCR reduction, as well as the percentage values of P/O ratio improvement compared to untreated samples, following Ataluren treatment at the lowest and highest doses and the shortest and longest incubation times in control (WT) cells, FANCA nonsense-mutated cells, and FANCA missense-mutated cells.**

For each metabolic readout, the table reports the percentage of reduction or improvement in samples treated either with 2.5 μM Ataluren for 24 h or with 10 μM Ataluren for 72 h, relative to the corresponding untreated samples, along with the p-value indicating statistical significance between the two measures (considered significant when p < 0.05).
Data are presented as mean ± standard deviation and are representative of three independent experiments.
